# Correlation between sestrin2 expression and airway remodeling in COPD

**DOI:** 10.1186/s12890-020-01329-x

**Published:** 2020-11-16

**Authors:** Da-Wei Zhang, Yuan-Yuan Wei, Shuang Ji, Guang-He Fei

**Affiliations:** 1 Department of Respiratory and Critical Care Medicine, First Affiliated Hospital of Anhui Medical University, Hefei, 230022 Anhui Province People’s Republic of China; 2 Key Laboratory of Respiratory Diseases Research and Medical Transformation of Anhui Province, Hefei, 230022 Anhui Province People’s Republic of China

**Keywords:** Sestrin2, Airway remodeling, COPD, Matrix metalloproteinase 9, Quantitative computed tomography

## Abstract

**Background:**

Airway remodeling is a major pathological characteristic of chronic obstructive pulmonary disease (COPD), and has been shown to be associated with oxidative stress. Sestrin2 has recently drawn attention as an important antioxidant protein. However, the underlying correlation between sestrin2 and airway remodeling in COPD has yet to be clarified.

**Methods:**

A total of 124 subjects were enrolled in this study, including 62 control subjects and 62 COPD patients. The pathological changes in airway tissues were assessed by different staining methods. The expression of sestrin2 and matrix metalloproteinase 9 (MMP9) in airway tissues was monitored by immunohistochemistry. Enzyme-linked immunosorbent assays (ELISAs) were used to detect the serum concentrations of sestrin2 and MMP9. The airway parameters on computed tomography (CT) from all participants were measured for evaluating airway remodeling. The relationship between serum sestrin2 and MMP9 concentration and airway parameters in chest CT was also analyzed.

**Results:**

In patients with COPD, staining of airway structures showed distinct pathological changes of remodeling, including cilia cluttered, subepithelial fibrosis, and reticular basement membrane (Rbm) fragmentation. Compared with control subjects, the expression of sestrin2 and MMP9 was significantly increased in both human airway tissues and serum. Typical imaging characteristics of airway remodeling and increased airway parameters were also found by chest CT. Additionally, serum sestrin2 concentration was positively correlated with serum MMP9 concentration and airway parameters in chest CT.

**Conclusion:**

Increased expression of sestrin2 is related to airway remodeling in COPD. We demonstrated for the first time that sestrin2 may be a novel biomarker for airway remodeling in patients with COPD.

**Supplementary Information:**

The online version contains supplementary material available at 10.1186/s12890-020-01329-x.

## Background

Chronic obstructive pulmonary disease (COPD) is the most common chronic airway inflammatory disease, and is characterized by persistent respiratory symptoms and irreversible airflow limitation. The pathological changes that contribute to the loss of lung function in COPD consist of parenchymal destruction (described clinically as emphysema), remodeling of the airways (described clinically as small airways disease), and mucus hypersecretion (described clinically as chronic bronchitis [1] . Airway remodeling of COPD mainly occurs in small airways (inner diameter < 2 mm), and is defined by changes in size, mass, and the quantity of airway structural components [1, 2].Functionally, airway remodeling produces airway obstruction and airway hyperactivity and reduces lung compliance [2], leading to poor clinical outcomes [3] . Thus, it is necessary to find possible biomarkers involved in airway remodeling in COPD.

Patients with COPD suffer an excessive oxidant burden due to tobacco, air pollution, and other harmful particles [1, 4]. Excessive oxidative stress contributes to enhanced airway remodeling and inversely correlates with lung function in patients with COPD [5, 6].Sestrins, a family of highly conserved proteins, are induced by oxidative and persistent hypoxia and expressed in the lung, brain, liver, heart and many other organs [7, 8]. Sestrin2, a typical member of the sestrin family, is the main component of the systemic antioxidant defense mechanism. Recent studies have shown that sestrin2 is involved in many oxidative stress-related respiratory diseases [9–11], including COPD. Previous studies on sestrin2 in COPD have primarily focused on animal pulmonary emphysema. It was reported that sestrin2 is upregulated and can regulate platelet-derived growth factor receptor beta (PDGFRβ) signaling or transforming growth factor β(TGF-β) signaling to regulate pulmonary emphysema in mouse models of COPD [12, 13]. However, human tissue can better reflect the real pathological characteristics of the occurrence and development of this disease. Unfortunately, these are hard to obtain. In this study, human airway tissue and serum were collected to study the correlation between sestrin2 and airway remodeling in patients with COPD.

The main evaluation methods for airway remodeling in this study involved three methods: histology, biomarkers and imaging technology. Major pathological features of airway remodeling in COPD include epithelial-mesenchymal transition (EMT), enhanced collage deposition, extracellular matrix degradation and repair, cilia dysfunction, and inflammatory infiltration [14, 15]. There is considerable literature showing that matrix metalloproteinases (MMPs), a family of zinc-dependent proteolytic enzymes, are involved in respiratory tract remodeling, particularly MMP9 [16, 17]. Previous studies have demonstrated that MMP9 was associated with pulmonary function and indicators of small airway disease in COPD [18, 19] .With the rapid development of imaging technology in recent years, CT has become useful for assessing airway dimensions and provides a valuable tool for the study of airway diseases [20]. Previous studies have found that quantitative CT-assessed parameters of the airway were correlated with pathological changes in airway remodeling [21, 22]. At present, there is still a lack of research on the relationship between sestrin2 and airway remodeling. The goal of this research was to investigate the expression characteristics of sestrin2 in patients with COPD and analyze the correlation between sestrin2 and airway remodeling.

## Methods

### .Subjects

Airway tissues were obtained from lobectomy or segmentectomy in four patients with lung cancer in situ and four COPD patients with comorbidity of lung cancer in situ at the First Affiliated Hospital of Anhui Medical University, China. Serum was obtained from a total of 124 participants (62 COPD patients and 62 controls) recruited from March 2018 to September 2019. The control group was collected from the physical examination center of this hospital during the same period. All patients with COPD met the diagnostic criteria of the 2018 GOLD guideline [23]. The exclusion criteria included patients with severe cardiovascular, hepatic and renal dysfunction, hematological system diseases, diabetes, obesity and malignant tumor; patients with mental illness; patients with other lung diseases (such as asthma, acute exacerbation of COPD, pneumonia, cystic fibrosis, active pulmonary tuberculosis, and interstitial lung disease); patients with a history of taking corticosteroids or immunosuppressants regularly; and patients with a history of participating in any health care activities before enrollment in this study.

### Pulmonary function tests

A pulmonary function test was performed on each subject at 15 min after inhaling 400 μg of salbutamol (Ventolin, GlaxoSmithKline, London, UK) using a dry spirometer device (Erich Jaeger GmbH, Hoechberg, Germany). Pulmonary function results were reported by experienced respiratory and critical care physicians. The following parameters were recorded: forced vital capacity (FVC), forced expiratory volume in one second (FEV1), FEV1% predicted, and the FEV1/FVC ratio. FEV1/FVC < 0.7 was classified as COPD.

### Chest computed tomography scan and analysis

The GE LightSpeed VCT (GE Healthcare, Milwaukee, US), GE Discovery C1750(GE Healthcare, Milwaukee, US), and Toshiba Aquilion 16-slice CT (Toshiba Medical Systems, Tokyo, Japan) were used for all scanning. All parameters were obtained by CT plain image reconstruction and were reconstructed using a standard algorithm. The lung window (window width 1500 HU, window level − 500 HU) and mediastinum window (window width 400 HU, window level 40 HU) were observed. Scanning parameters were as follows: GE LightSpeed VCT and GE Discovery C1750 machines used a tube voltage of 120 kV, automatic tube current regulation, a layer thickness of 5.0 mm, a reconstruction layer thickness 0.625 mm, and pitch 1.375; Toshiba Aquilion 16-layer CT instruments used a tube voltage of 120 kV, a tube current of 150 mA, a layer thickness 5.0 mm, a reconstruction layer thickness of 1.0 mm, and a pitch 0.980. Airway quantitative measurements were performed using the thoracic VCAR software supplied by GE in the apical segment of the right upper lobe (RB1). The following parameters could be automatically measured using the thoracic VCAR software: the square root of the wall area at an internal airway area of 8 mm^2^ (Ai8), the percentage of bronchial wall area (WA %), total airway area(A_O_), and the relative wall thickness (RWT) (expressed as the ratio of wall thickness and external diameter) [24].

### Measurement of serum sestrin2 and MMP9 concentrations

Blood samples were collected from all subjects by venipuncture with a tube without anticoagulants. The serum was collected after centrifugation for 20 min at 1000×g and then stored at − 80 °C. The serum sestrin2 and MMP9 concentrations were determined using human enzyme-linked immunosorbent assay (ELISA) kits following the manufacturer’s instructions. The ELISA kits were against sestrin2(Cloud-Clone Crop, Wuhan, China) and MMP9 (CUSABIO, Wuhan, China).

### Histological staining of airway tissues

Human lung tissues were fixed in 4% paraformaldehyde and then embedded in paraffin. Sections with a thickness of 5 μm were prepared for staining. Hematoxylin-eosin (HE), periodic Schiff-methenamine silver (PASM) and Masson staining kits (Solarbio, Beijing, China) were employed to test for structural changes of the human airway. The experimental methods followed the manufacturer’s instructions. Immunohistochemical staining was applied to detect the expression of sestrin2 and MMP9 in these sections. Antigens were retrieved according to primary antibody specifications. An endogenous peroxidase blocker was added and incubated for 30 min at 37 °C. Slides were then incubated with rabbit anti-sestrin2 antibody (ProteinTech#10795–1-AP, Chicago, USA; diluted 1:200) and mouse anti-MMP9 antibody (Abcam #ab58803, Cambridge, UK; diluted 1:100) at 4 °C overnight. Then, the slides were washed with PBS, followed by incubation with biotinylated goat anti-rabbit IgG and then incubated with streptavidin-peroxidase. Diaminobenzidine (DAB) solution (ZSGB-Bio, Beijing, China) was used for staining. Finally, all slides were counterstained with hematoxylin. Staining was imaged using a light microscope (Leica ICC50 W, Wetzlar, Germany). Semiquantitative assessment of MMP9 and sestrin2 protein expression was performed using Image-Pro Plus6.0 (Media Cybernetics, Inc., USA). The mean integral optical density of protein staining was the ratio of the integral optical density of protein stained-positive epithelium to the area of corresponding bronchial epithelium.

### Statistical analysis

GraphPad Prism 5 (San Diego, California, USA) and SPSS 22.0 (IBM, Armonk, NY, USA) software were used to analyze all data. Categorical variables were expressed as counts (%). A chi-square test was used to examine the differences between sex ratios and smoking status. Normally distributed data were compared using an unpaired t-test and expressed as the mean (plus or minus the standard deviation). All non-normally distributed data were compared using the Mann-Whitney U test and were expressed as the median with the interquartile range. The correlation between serum protein concentration and other measurement indices in the COPD group was analyzed using Pearson or Spearman rank correlation analysis. A *P*-value of less than 0.05 was regarded as statistically significant.

## Results

### Demographic characteristics of all subjects

All subjects recruited in this study were divided into two groups: a COPD group(*n* = 62) and a control group(n = 62). There were no significant differences in age, sex, BMI, or smoking index between these two groups. However, other pulmonary function indicators (FEV1, FEV1%, and FEV1/FVC) were significantly decreased in the COPD group (*P* < 0.001). The demographic characteristics of all subjects are listed in Table 1.

**Table 1:** Demographic characteristics of the control group and COPD group

Variables	Control group(***n*** = 62)	COPD group(***n*** = 62)	***P*** value
Age, years	67.37 (6.87)	68.73 (8.47)	0.330^a^
Mal sex, *n* (%)	34 (54.84%)	31 (50.00%)	0.590^c^
BMI, kg/m^2^	22.90 (3.32)	22.17 (4.29)	0.826^b^
Smoking status			0.234^c^
Never smoked, *n* (%)	50 (80.65%)	45 (72.58%)	
Current smoker, *n* (%)	8 (12.90%)	7 (11.29%)	
Ex-smoker, *n* (%)	4 (8.06%)	10 (16.13%)	
Smoking, pack-years*	29.38 (15.16)	31.94 (17.87)	0.689^a^
Pulmonary function			
FEV1, L	2.84 (0.66)	1.06 (0.55)	< 0.001^a^
FEV1/FVC, %	82.95 (7.19)	59.58 (7.76)	< 0.001^b^
FEV1, % of predicted	104.30 (12.58)	38.00 (21.20)	< 0.001^b^

**Notes:** Data are presented as number (%) or means (standard deviation) or median (interquartile range). *(number of cigarettes per day × number of years of smoking) /20

***Abbreviations*:** *COPD* chronic obstructive pulmonary disease, *BMI* body mass index, *FEV1* forced expiratory volume in 1 s, *FVC* forced vital capacity, ^*a*^*t-test* ^b^Mann-Whitney U test; ^c^χ2 test

### Histological staining of airway tissues

**Fig. 1:**
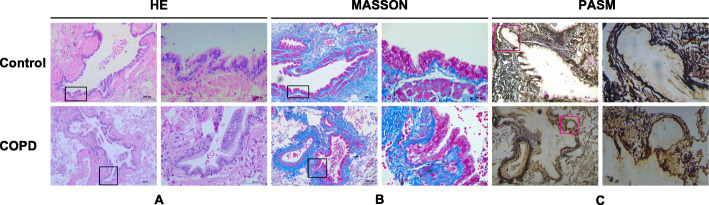
Staining of airway tissues in control and COPD group. Notes: **a** H&E stains in control and COPD group; **b** Subepithelial fibrosis by Masson staining in control and COPD group; **c** Change of reticular basement membrane thickness by PASM staining in control and COPD group. Magnification, × 400

To characterize airway remodeling at the pathological level, airway tissues were isolated and various staining methods were then used. HE staining demonstrated that the airway epithelial structure and ciliated cells were intact in the control group, while the ciliated cells were detached and lodged to different degrees in the COPD group (Fig. 1a). Masson staining showed that collagen deposition in the bronchial epithelial area of the COPD group was significantly increased compared with the control group (Fig. 1b). Continuous Rbm was clearly observed in the control group, but the reticular basement membrane was fragmentary in the COPD group based on PASM staining (Fig. 1c).

### Expression of sestrin2 and MMP9 in airway tissues

**Fig. 2:**
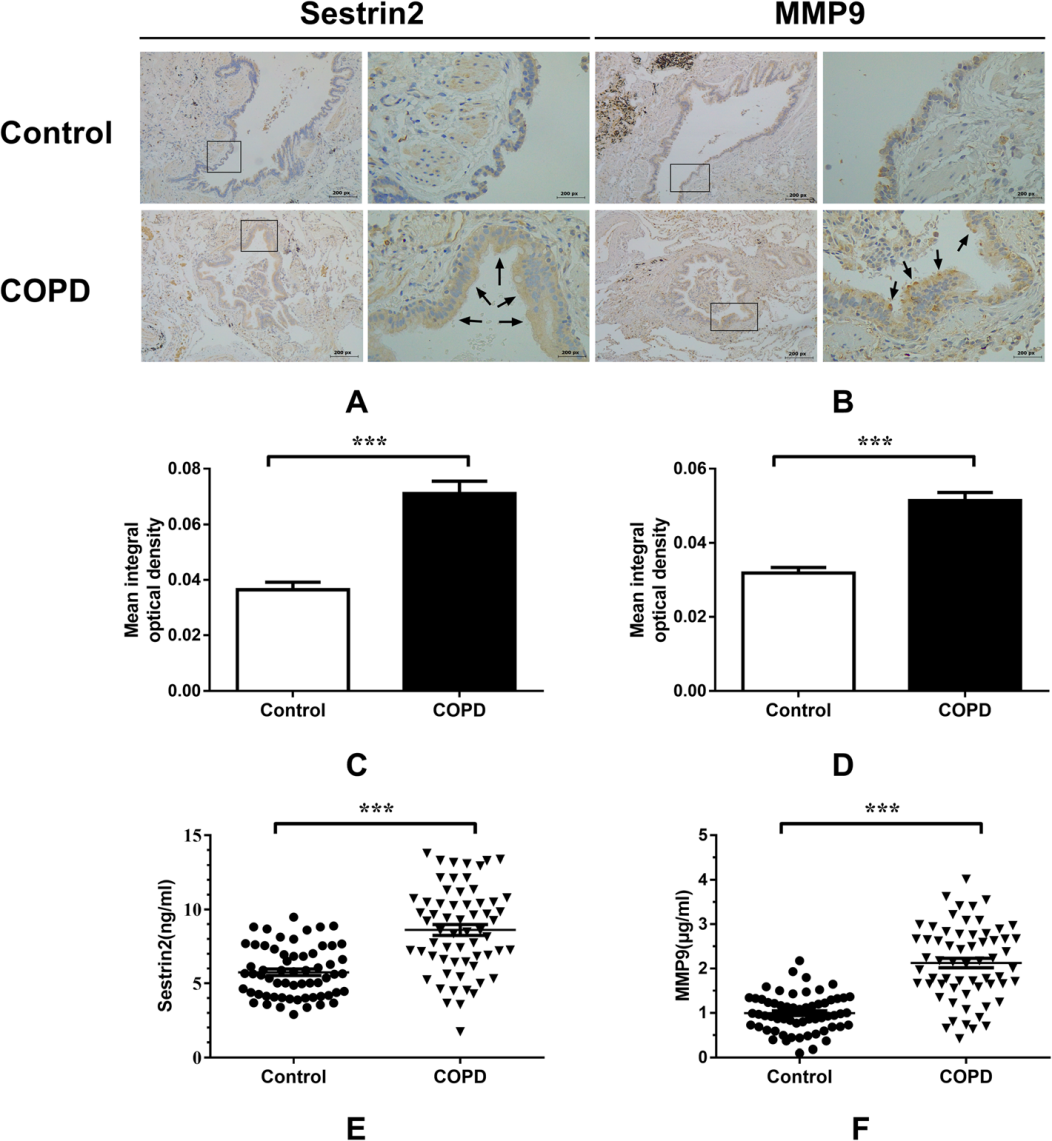
Sestrin2 and MMP9 expression in airway tissue and serum in control and COPD group. Notes: **a** Expression of sestrin2 by IHC in control and patients with COPD. **b** Expression of MMP9 by IHC in control and patients with COPD. **c** Semiquantitative assessment of sestrin2 expression using Image-Pro Plus. **d** Semiquantitative assessment of MMP9 expression using Image-Pro Plus. **e** Serum sestrin2 concentrations in control and COPD groups. **f** Serum MMP9 concentrations in control and COPD groups; positive protein staining appears brown and nuclear staining appears blue. Magnification, × 400. *P < 0.05, **P < 0.01, ***P < 0.001 versus control group

Brown staining represents positive protein expression in immunohistochemical analysis using DAB. As described previously [25, 26], sestrin2 expression was largely located in the cytoplasm of bronchial epithelial cells and was significantly increased in the COPD group (Fig. 2a). Stronger MMP9 expression was also clearly detected in the cytoplasm in the COPD group when compared with the control group (Fig. 2b). The mean integral optical densities of sestrin2 and MMP9 were significantly higher in the COPD group compared with the control group (*P* < 0.001; Fig. 2c-d).

### Serum concentrations of sestrin2 and MMP9

Compared with the control group, the serum sestrin2 concentration was significantly increased in patients with COPD(*P* < 0.001) (Table 2, Fig. 2e). Similarly, the expression of MMP9 in serum was significantly higher in patients with COPD than in controls(*P* < 0.001) (Table 2, Fig. 2f). There was no difference between smokers and non-smokers in terms of serum senestrin2 and MMP9 concentration (see Additional file 1). There was also no difference between the different sexes in COPD patients (see Additional file 2).

**Table 2:** Serum sestrin2 and MMP9 concentrations and quantitative CT measurements in the total subjects

Test index	Control group(***n*** = 62)	COPD group(***n*** = 62)	***P*** value
Serum assay			
Sestrin2(ng/ml)	5.00 (3.93)	8.61 (2.89)	< 0.001^b^
MMP9(ng/ml)	993.02 (421.30)	2125.65 (840.81)	< 0.001^a^
Airway parameters on chest CT			
Ai8(mm)	4.45 (0.28)	5.51 (0.64)	< 0.001^a^
A_O_ (mm^2^)	28.05 (2.50)	38.72 (7.41)	< 0.001^a^
WA% (%)	70.53 (3.01)	78.31 (3.73)	< 0.001^a^
RWT	0.23 (0.11)	0.27 (0.02)	< 0.001^a^

**Notes:** Data are presented as means (standard deviation) or median (interquartile range). *P*-values were calculated by statistical analysis of variable

***Abbreviations*:** *CT* computed tomography, *MMP9* matrix metalloproteinases 9, *Ai8* Square root of the wall area at an internal airway area of 8 mm^2^, *A*_*O*_ total airway area, *WA%* wall area percentage, *RWT* the ratio of airway wall thickness to overall diameter, ^*a*^*t-test* ^b^Mann-Whitney U test

### Airway parameters in chest CT scans of subjects

**Fig. 3:**
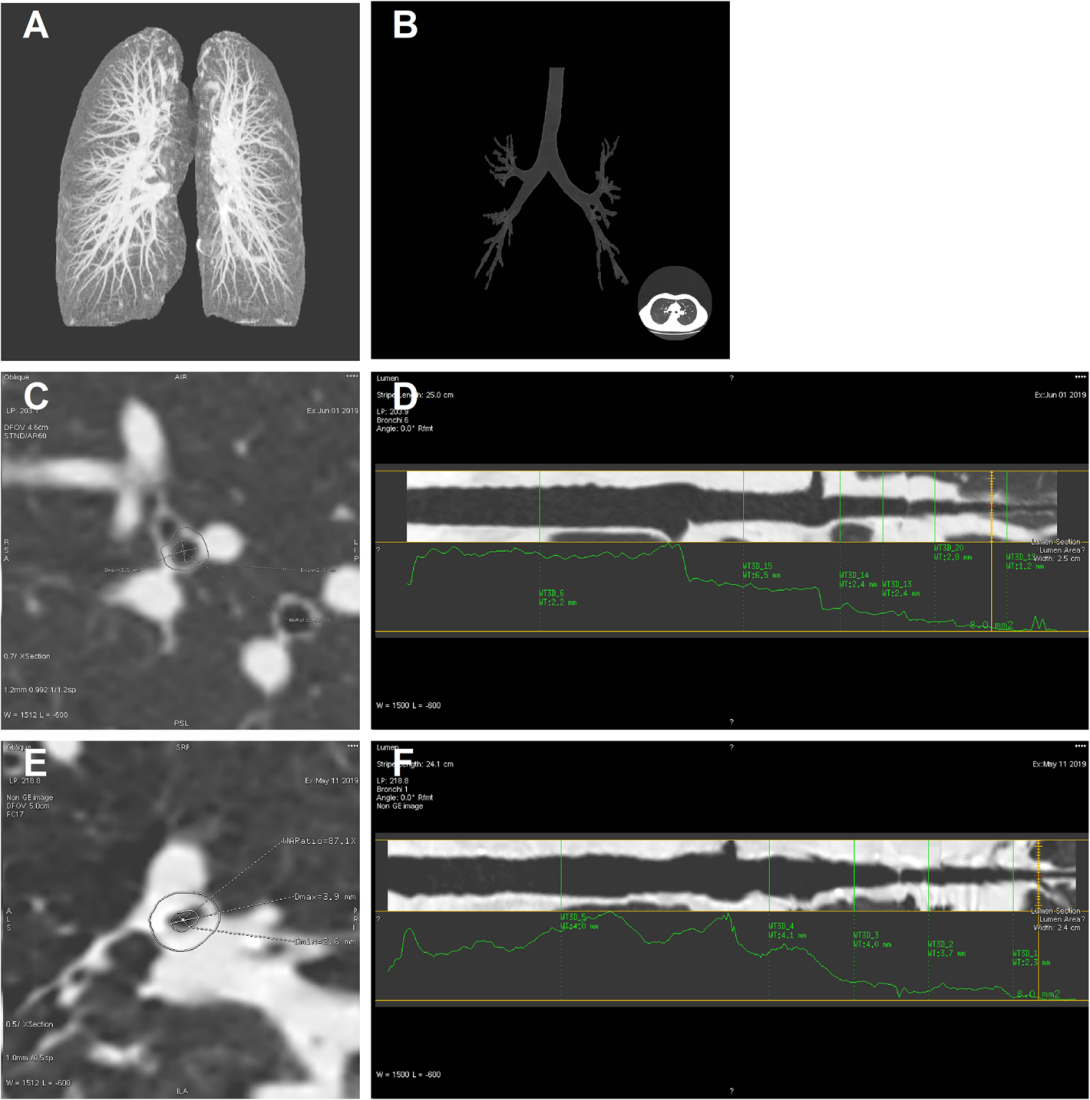
Chest computed tomography image in control and COPD group. Notes: **a** Lung tissue extracted automatically by software by Thoracic VCAR software; **b** Airway tree extracted automatically by software by Thoracic VCAR software; **c** & **e** Cross-section of an exact airway with lumen area 8mm^2^, shows delineation of outer and inner bronchial wall, permitting calculation of airway measurements in control and COPD groups; **d** & **f** Curved planar reformation of the bronchial pathway in control and COPD group

**Fig. 4:**
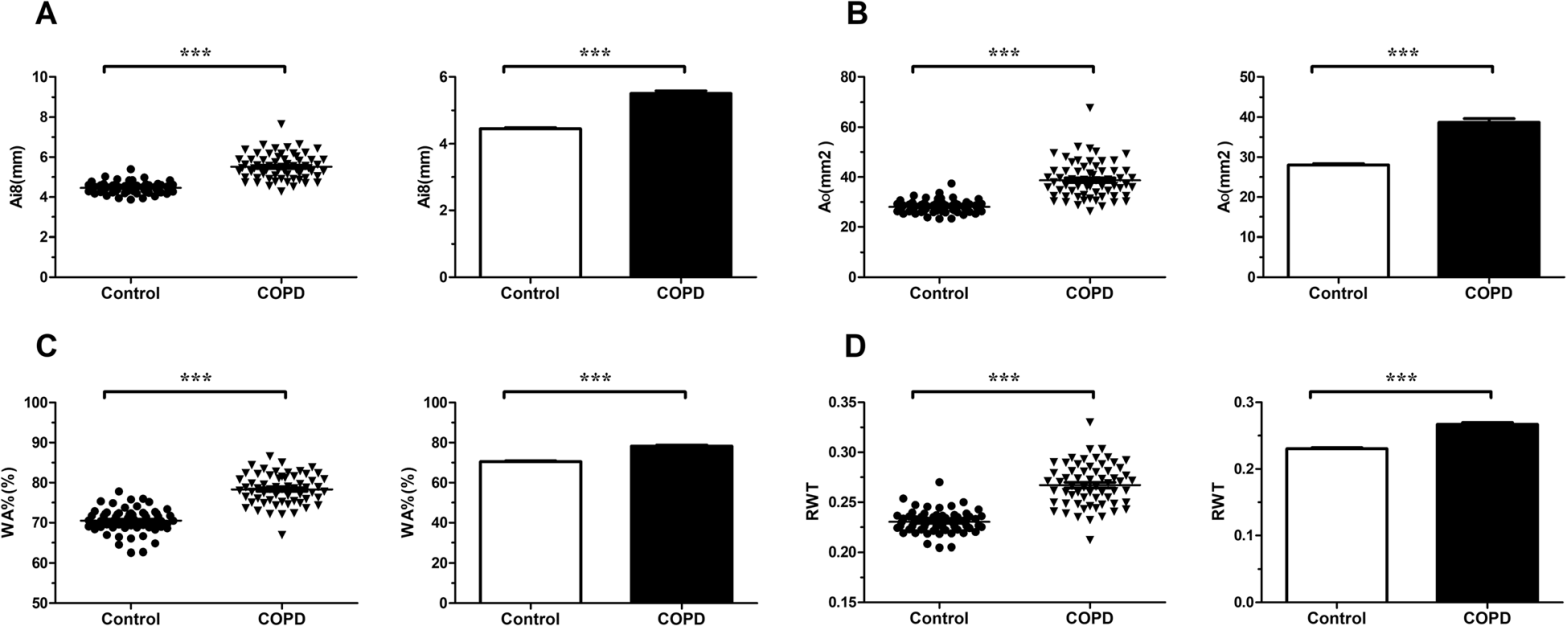
Airway parameters on chest CT in control and COPD group. Notes**: a** Ai8 in control and COPD group; **b** A_O_ in control and COPD group; **c** WA% in control and COPD group; **d** RWT in control and COPD group. *P < 0.05, **P < 0.01, ***P < 0.001 versus control group

Compared with the control group, chest CT imaging showed that the bronchial wall was thickened, and the bronchial lumen was rough and curved in patients with COPD(Fig. 3). We demonstrated that the values of airway parameters in chest CT (Ai8, A_O_, WA%, and RWT) were all significantly increased in the COPD group(*P* < 0.001) (Table 2, Fig. 4).

### Relationship between sestrin2 and MMP9, CT parameters, and pulmonary function in patients with COPD

**Fig. 5:**
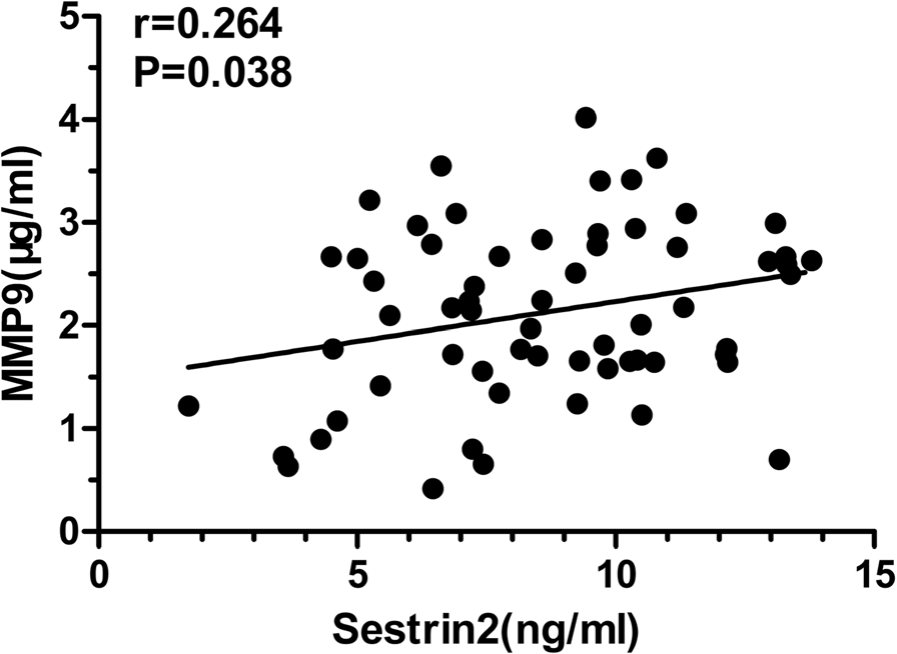
Correlation between serum sestrin2 concentrations and serum MMP9 concentrations. Notes: Correlations between serum sestrin2 concentrations and serum MMP9 concentrations. Correlations were determined by Pearson rank correlation analysis

**Fig. 6:**
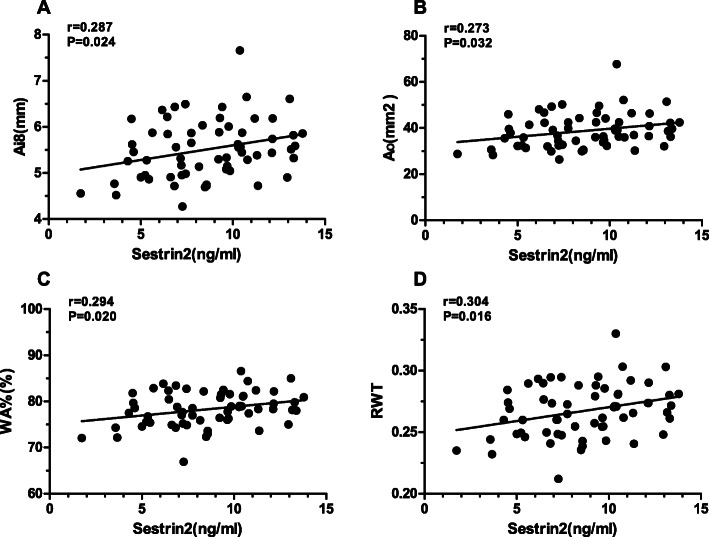
Correlation between serum sestrin2 concentrations and airway parameters on chest CT. Notes: Correlations between serum sestrin2 concentrations and Ai8 **a**, A_O_ **b**, WA% **c**, and RWT **d**. Correlations were determined by Pearson rank correlation analysis

To elucidate the relationship between sestrin2 and airway remodeling in patients with COPD, the correlation between serum sestrin2 concentration and MMP9, as well as airway parameters in chest CT, were analyzed in patients with COPD. The results showed that the serum sestrin2 concentration was positively correlated with serum MMP9 concentration (r = 0.264;*P* = 0.038) (Fig. 5), Ai8 (r = 0.287;*P* = 0.024) (Fig. 6a), A_O_(r = 0.273;*P* = 0.032) (Fig. 6b),WA% (r = 0.294;*P* = 0.020) (Fig. 6c) and RWT (r = 0.304;*P* = 0.016) (Fig. 6d). However, there were no significant associations between serum sestrin2 concentration and parameters of lung function (Table 3).

**Table 3:** Association between serum sestrin2 concentrations and other measurement indices in COPD group

Test index	r	***P*** value
Serum assay		
MMP9(ng/ml)	0.264	0.038^a^
Airway parameters on chest CT		
Ai8(mm)	0.287	0.024 ^a^
A_O_ (mm^2^)	0.273	0.032 ^a^
WA% (%)	0.294	0.020 ^a^
RWT	0.304	0.016 ^a^
Pulmonary function		
FEV1, L	0.044	0.732^b^
FEV1/FVC, %	−0.099	0.444^b^
FEV1, % of predicted	0.118	0.359^b^

**Notes:** Correlations were determined by Pearson or Spearman rank correlation analysis

***Abbreviations*:** *CT* computed tomography, *MMP9* matrix metalloproteinases 9, *Ai8* Square root of the wall area at an internal airway area of 8 mm^2^, *Ao* total airway area, *WA%* wall area percentage, *RWT* a ratio of airway wall thickness to overall diameter, *FVC* forced vital capacity, *FEV1* forced expiratory volume in one second, *a* Pearson rank correlation analysis, *b* Spearman rank correlation analysis, *r* Pearson or Spearman rank correlation coefficient

### Relationship between MMP9 and CT parameters in patients with COPD

There was no association between serum MMP9 concentration and Ai8 (r = 0.123; *P* = 0.340), A_O_ (r = 0.144; *P* = 0.264), WA% (r = 0.087; *P* = 0.500) and RWT (r = 0.128; *P* = 0.323), as shown in Additional file 3.

## Discussion

The goal of this study was to determine the possible relationship between sestrin2 and airway remodeling in COPD. At the histological level, immunohistochemical staining of human airway tissues demonstrated that expression of sestrin2 and MMP9 was much higher in the COPD group than that in the control group. At the biomarker level, the serum concentrations of sestrin2 and MMP9 were increased in patients with COPD. At the imaging level, the airway structure showed visible imaging characteristics of airway remodeling and quantitative airway parameters in chest CT were significantly increased in patients with COPD. We further demonstrated that the serum sestrin2 level correlated well with the serum MMP9 level and quantitative airway parameters in chest CT. Thus, this study demonstrated that sestrin2 might participate in airway remodeling in COPD.

Airway remodeling is a recognized characteristic in the course of COPD [27], which is a complex structural change caused by multiple factors [16, 28, 29]. Our study demonstrated that there are typical pathological characteristics of airway remodeling in the airway tissues of COPD, including airway subepithelial fibrosis, reticular basement membrane fragmentation and cilia clutter. MMP9, a matrix metalloproteinase, has often been described as an indicator of airway remodeling in COPD [30]. Consistent with previous reports [22, 30, 31], the expression of MMP9 in airway tissue and serum was significantly increased in the COPD group in our study. These findings showed that significant airway remodeling was present in patients with COPD.

Given that oxidative stress is an important mechanism for airway remodeling, our study demonstrated that the concentration of sestrin2 in the COPD group was significantly increased in human serum compared with the control group. Sestrin2, a critical antioxidant protein, plays a protective role in antioxidant defense and functions to reduce intracellular reactive oxygen species (ROS) [32–35]. Emerging evidence have shown that sestrin2 plays a vital role in the formation and development of fibrosis in many organs, including the heart, liver and kidney [8, 36–38]. To further explore whether sestrin2 is related to airway remodeling in COPD, immunohistochemical staining in the airway was done and showed that there was a significantly higher expression of sestrin2 in the airway in the COPD group compared with the control group, especially in the bronchial epithelial cells, which are replete with MMP9. It was reported that sestrin2 knockdown increases Lipopolysaccharides(LPS)-mediated expression of MMP9 in heart tissue [39]. In our study, we also found that the serum sestrin2 concentrations were positively correlated with serum MMP9 concentrations. Therefore, these findings highlight the possible utility of sestrin2 as a blood biomarker for airway remodeling in COPD.

Given that airway parameters in chest CT have been regarded as radiological biomarkers for COPD and are associated with airflow obstruction in all GOLD stages [40–42], airway structure and airway parameters in chest CT were obtained to assess airway remodeling in our study. We demonstrated that the COPD group showed apparent changes in the airway structure, characterized by a thickened bronchial wall and rough and curved bronchial lumen. The four quantitative airway parameters in chest CT (Ai8, A_O_, WA%, and RWT) were all 9statistically increased, which demonstrated airway remodeling in COPD at the imaging level. It was reported that there was a significant correlation of sputum elastase with the thickness of bronchial wall as measured by chest CT [43]. In this study, we demonstrated that there was a positive relationship between serum sestrin2 concentration and quantitative airway parameters in chest CT. However, we have found no significant associations between MMP9 and quantitative CT measurements, consistent with Górka [44]. O’Donnell [45] considered that bronchial wall thickening was not directly linked to luminal inflammatory indices or protease activity and the remodeling in the proximal airway may be more related to inflammatory infiltration of the submucosa. These findings suggested that sestrin2 may better reflect the airway remodeling of COPD.

In our study, we did not find the correlations between sestrin2 and parameters of lung function. There have been no studies on the correlation between sestrin2 and lung function in COPD to date. Larger sample sizes, however, are needed to verify these results. The present study also had certain limitations. First, due to the fact most patients with mild COPD are unaware of their condition or never receive regular lung function tests, our subjects were mostly patients with moderate to severe COPD. Furthermore, sestrin2 levels have been reported to decline after treatment in Parkinson’s disease, asthma and obstructive sleep apnea [9, 11, 46]. We did not follow up on the changes in serum sestrin2 and MMP9 concentrations or quantitative airway parameters in chest CT in patients with COPD after regular treatment.

## Conclusions

In summary, the airway of patients with COPD showed significant airway remodeling at the histology, biomarker and imaging levels. The expression of sestrin2 was significantly increased in patients with COPD. A positive correlation was found in serum sestrin2 concentration and MMP9 concentration as well as quantitative airway parameters in chest CT as markers of airway remodeling. Sestrin2 may serve as a potential clinical biomarker for airway remodeling in COPD in the future.

## Supplementary Information

**Additional file 1 **: **Table S1.** Serum sestrin2 concentration in subjects with different smoking status.

**Additional file 2 **: **Table S2.** Serum sestrin2 concentration in different sex in COPD patients.

**Additional file 3 **: **Table S3.** Association between serum MMP9 concentration and airway parameters in chest CT in COPD group.

## Data Availability

The datasets used and/or analyzed during the current study available from the corresponding author on reasonable request.

## Abbreviations

COPD: Chronic obstructive pulmonary disease
MMP9: Matrix metalloproteinase 9
ELISA: Enzyme-linked immunosorbent assay
CT: Computed tomography
Rbm: Reticular basement membrane
EMT: Epithelial-mesenchymal transition
MMPs: Matrix metalloproteinases
FVC: Forced vital capacity
FEV1: Forced expiratory volume in one second
Ai8: Square root of the wall area at an internal airway area of 8 mm2
WA %: The percentage of bronchial wall area
A_O_: Total airway area
RWT: Relative wall thickness
DAB: Diaminobenzidine
ROS: Reactive oxygen species
LPS: Lipopolysaccharides
IHC: Immunohistochemical
HE: Hematoxylin-eosin
ASM: Periodic Schiff-methenamine silver
PDGFRβ: Platelet-derived growth factor receptor beta
TGF-β: Transforming growth factor β
