# Supplementary material for: Correlation between sestrin2 expression and airway remodeling in COPD
Source: BMC Pulm Med. 2020 Nov 16;20:297. doi: 10.1186/s12890-020-01329-x (PMC7667887; doi:10.1186/s12890-020-01329-x)
Supplement: Supplementary file 1 — Additional file 1 : Table S1. Serum sestrin2 concentration in subjects with different smoking status. [file 12890_2020_1329_MOESM1_ESM.docx]

**Table S1: Serum sestrin2 concentration in subjects with different smoking status**

| **Test index** | **Smokers(n=95)** | **Non-smokers(n=29)** | ***P* value** |
| --- | --- | --- | --- |
| Sestrin2(ng/ml) | 7.73(3.05) | 7.02 (2.67) | 0.183^a^ |

**Notes:** Data are presented as means (standard deviation) or median (interquartile range). *P*-values were calculated by t-test.

**Abbreviations:** ^a^t-test；
