# Supplementary material for: Correlation between sestrin2 expression and airway remodeling in COPD
Source: BMC Pulm Med. 2020 Nov 16;20:297. doi: 10.1186/s12890-020-01329-x (PMC7667887; doi:10.1186/s12890-020-01329-x)
Supplement: Supplementary file 2 — Additional file 2 : Table S2. Serum sestrin2 concentration in different sex in COPD patients. [file 12890_2020_1329_MOESM2_ESM.docx]

**Table S2: Serum sestrin2 concentration in different sex in COPD patients**

| **Test index** | **Male(n=31)** | **Female(n=31)** | ***P* value** |
| --- | --- | --- | --- |
| Sestrin2(ng/ml) | 8.31(3.06) | 8.92 (2.72) | 0.249^a^ |

**Notes:** Data are presented as means (standard deviation) or median (interquartile range). *P*-values were calculated by t-test .

**Abbreviations:** ^a^t-test；
