# Supplementary material for: Correlation between sestrin2 expression and airway remodeling in COPD
Source: BMC Pulm Med. 2020 Nov 16;20:297. doi: 10.1186/s12890-020-01329-x (PMC7667887; doi:10.1186/s12890-020-01329-x)
Supplement: Supplementary file 3 — Additional file 3 : Table S3. Association between serum MMP9 concentration and airway parameters in chest CT in COPD group. [file 12890_2020_1329_MOESM3_ESM.docx]

**Table S3: Association between serum MMP9 concentration and airway parameters in chest CT in COPD group**

| **Test index** | **r** | ***P* value** |
| --- | --- | --- |
| Ai8(mm) | 0.123 | 0.340^a^ |
| A_O_ (mm^2^) | 0.144 | 0.264^a^ |
| WA% (%) | 0.087 | 0.500^a^ |
| RWT | 0.128 | 0.323^a^ |

**Notes:** Correlations were determined by Pearson or Spearman rank correlation analysis.

**Abbreviations:** MMP9, matrix metalloproteinases 9; Ai8, Square root of the wall area at an internal airway area of 8 mm^2^; Ao, total airway area; WA%, wall area percentage; RWT, a ratio of airway wall thickness to overall diameter; a, Pearson rank correlation analysis;b, Spearman rank correlation analysis
